# Chitosan-Derived Porous Activated Carbon for the Removal of the Chemical Warfare Agent Simulant Dimethyl Methylphosphonate

**DOI:** 10.3390/nano9121703

**Published:** 2019-11-28

**Authors:** Hyejin Yu, Ye Rim Son, Hyeonji Yoo, Hyun Gil Cha, Hangil Lee, Hyun Sung Kim

**Affiliations:** 1 Department of Chemistry, Pukyong National University, Busan 48513, Korea; yuhj93@krict.re.kr (H.Y.); syl75218@daum.net (Y.R.S.); hjyoo1996@gmail.com (H.Y.); 2 Center for Bio-based Chemistry, Korea Research Institute of Chemical Technology (KRICT), Ulsan 44429, Korea; 3 Department of Chemistry, Sookmyung Women’s University, Seoul 04310, Korea

**Keywords:** activated carbon, chitosan, adsorption, chemical warfare

## Abstract

Methods for the rapid removal of chemical warfare agents are of critical importance. In this work, a porous activated carbon material (C-PAC) was prepared from chitosan flakes via single-step potassium carbonate (K_2_CO_3_) activation for the prompt adsorption of dimethyl methylphosphonate (DMMP). C-PAC samples were prepared using different carbonization temperatures (350, 550, and 750 °C) at a constant K_2_CO_3_/chitosan ratio (1:2) and using different activator ratios (K_2_CO_3_/chitosan ratios of 1:0.5, 1:1, 1:2, and 1:3) at 750 °C. Furthermore, we evaluated the effect of preparation conditions on the adsorption capacities of the various C-PAC materials for DMMP under ambient conditions (25 °C). Notably, for the C-PAC material prepared at 750 °C using a K_2_CO_3_/chitosan ratio of 1:2, the DMMP adsorption was saturated at approximately 412 mg·g^−1^ carbon after 48 h. The good performance of this material makes it a potential candidate for use in remedial applications or protective gear.

## 1. Introduction

Regulations related to the development, production, stockpiling, and usage of chemical weapons are continuously being made more stringent to protect human society. Nerve agents are one of the most toxic classes of chemical warfare agents (CWAs) [1,2]. In particular, organophosphorus molecules possessing P–X bonds (X = F, CN, and SR) inhibit acetylcholinesterase, the key enzyme involved in neuronal signaling [3]. Despite the international ban on their use, various nerve agents are still deployed in modern chemical warfare. Thus, the development of methods or materials for the rapid detoxification or removal of CWAs from the environment is critical. The decontamination of CWAs is arguably the most challenging issue facing militaries around the world. Furthermore, owing to possible terror threats, protecting civilians from CWAs has become increasingly important for many governments. The prompt adsorptive removal of toxic agents from various environments is one of the most critical technologies for achieving the rapid detoxification of CWAs [4,5,6].

Among various candidate solid materials, adsorbents based on activated carbon have been presented numerically to be effective and practical materials for the adsorption and desorption of toxic and persistent molecules, owing to their high surface area, easy chemical modification, corner defect sites resulting from unusual lattice planes, low economic cost, and reproducibility [7,8]. Many studies have focused on methods to synthesize activated carbons with a high surface area, large pore volume, and small crystal size to improve the sorption capacity and reactivity for toxic chemicals. Currently, there is considerable interest in developing inexpensive and effective alternatives to existing commercial activated carbons, which will enhance environmental sustainability and offer various benefits for future commercial applications. The preparation of activated carbons from biomaterials such as wood [9,10], peat [11], coconut [12] and etc. [13,14] is expected to be economical. In particular, chitosan, a biopolysaccharide composed primarily of 2-amino-2-deoxy-D-glucose units and derived from chitin in crustacean cells, insect exoskeletons, and fungus cell walls, is the second most abundant biopolymer after cellulose. Because chitosan is naturally rich in nitrogen and possesses specific acid–base properties, there are various reports on the preparation of carbon materials using chitosan as a carbon precursor [15,16,17,18,19,20,21,22,23]. Kucinska et al. reported a practical method for converting chitosan into activated carbon by employing a Na_2_CO_3_ solution as an activator agent, which gave a porous carbon with a low surface area (400 m^2^·g^−1^) [22]. Another common method for obtaining activated carbons involves dissolution of chitosan in dilute acetic acid followed by hydrothermal carbonization at a mild temperature. Furthermore, a few studies have focused on the synthesis of chitosan-derived activated carbons by a chemical activation process using KOH as an activation agent [23].

Herein, porous activated carbon materials were derived from chitosan by employing K_2_CO_3_ as a mild activator. The texture of the porous carbons obtained following chitosan carbonization was easily regulated by varying the K_2_CO_3_ to chitosan ratio and the activation temperature. To exemplify the applicability of the prepared porous activated carbon materials as efficient adsorbents for CWAs, the adsorptive removal of dimethyl methylphosphonate (DMMP) as a nerve agent simulant was investigated.

## 2. Materials and Methods

### 2.1. Preparation of Activated Chitosan Carbon

The desired amount of K_2_CO_3_ was completely dissolved in DDW (10 mL) in a glass vial. Chitosan (3 g) was added and the mixture was stirred for 30 min. The above aqueous mixture was dried at 100 °C in an oven overnight. A dried chitosan solid mixture was ground into fine powder using mortar. The fine powder sample was placed inside in a quartz tube furnace. Carbonization was performed at the desired temperature under Ar flow for 2 h (5 °C/min heating rate). After cooling to room temperature, etching with 0.1 M HCl for 1 h under ultrasonication was performed to remove remaining K_2_CO_3_ and carbonized chitosan was dried at 100 °C in an oven for overnight. The obtained materials were denoted by C-PAC t (1:*x*). (*t* = carbonization temperature, *x* = relative amount of K_2_CO_3_)

### 2.2. Adsorption of DMMP on Activated Carbon Derived from Chitosan

Carbons were pretreated in a vacuum oven at 100 °C for ≥6 h under static conditions to remove low volatile adsorbed contaminants before being exposed to DMMP. Approximately 25 mg carbon was transferred to small vials and placed inside larger vials with liquid DMMP, capped, and stored in a desiccator. The exposure process was conducted at atmospheric pressure and room temperature (24–26 °C). Samples were removed and analyzed at desired exposure times using a thermogravimetric analysis (TGA).

### 2.3. Desorption of DMMP from Activated Carbon Derived from Chitosan

All lines downstream of the reactor were maintained at a constant 200 °C before testing to remove contaminated other organic molecules adsorption in reaction lines. Then, the effective adsorption capacity measurements were programmed to include the following temperature regimes: (1) A linear temperature increase of 10 °C min^−1^, from 60 to 400 °C. (2) Hold at 400 °C for 10 min. (3) A fast cooling to the starting temperature. To trap desorbed DMMP, the reactor effluent was bubbled through cold trap contained acetonitrile with *N*,*N*-dimethyl methanamide(DMF) as internal standard maintained at 0 °C via an ice/water bath. The sample was analyzed using a gas chromatography (GC) analysis to quantify the DMMP content desorbed from the carbon sample upon heating.

### 2.4. Instrumentation

X-ray diffraction (XRD) measurements were performed using an Ultima IV Rigaku analyzer (Rigaku Corp., Tokyo, Japan) equipped with an X-ray tube (40 kV, 30 mA) using Cu Kα radiation (Kα = 1.54056 A). Raman spectroscopic measurements were performed using a Thermo Fisher DXR Raman microscope (Thermo Fisher Scientific, Waltham, MA, USA) equipped with green laser (532 nm) as the excitation source. Fourier-transform infrared (FTIR) spectra were recorded on IR-4000 (JASCO International Co., Ltd., Tokyo, Japan) in the region of 4000 to 400 cm^−1^. The samples were prepared as KBr pellets. Field emission scanning electron microscope (FE-SEM) measurements were performed by using a TESCAN Mira 3 (TESCAN, Kohoutovice, Czech Republic) operated at 20 kV. X-ray photoelectron spectroscopy (XPS) studies were carried out using an AXIS SUPRA spectrometer (KRATOS Analytical Ltd., Manchester, UK) using an Al Kα excitation source with a photon energy of 1500 eV. Low-pressure gas sorption isotherms were collected on a BELSORP-max (BEL Japan Inc., Tokyo, Japan). Prior to the measurements, all carbon samples were pretreated by evacuate under vacuum (~20 mTorr) at 200 °C for 12 h. Then, their surface area, total pore volume, and micropore volume were obtained from the N_2_ adsorption isotherm at 77 K. For the quantitative analysis, we used TGA and gas chromatography (GC). The TGA were performed using TA Instrument TGA Q 500 (TA Instruments, New Castle, DE, USA). Samples (~2 mg) were placed in a platinum pan. The TGA were performed in a temperature range of 30~450 °C with a high purity N_2_ purge gas flow (40 mL/min). A GC instrument, model 5890 (Agilent Technologies, Inc., Santabarbara, CA, USA) equipped with a flame ionization detector (FID) and a capillary polar column (DB-5, Agilent Technologies, Inc., Santabarbara, CA, USA) was used to quantitatively determine the adsorption amounts of DMMP.

## 3. Results

### 3.1. Chatacterization of C-PAC Samples

**Figure 1:**
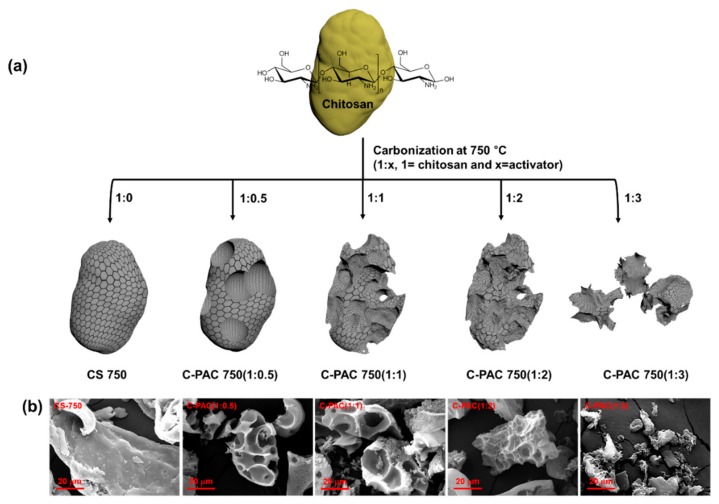
(**a**) Illustrations of morphologies and (**b**) SEM images of activated carbons prepared by carbonization of chitosan at various activator ratios.

Using the procedure described in the Experimental section, we synthesized chitosan-derived porous carbon samples. The microstructural morphologies of the as-synthesized samples obtained at different temperature and activator to chitosan ratios were characterized by FE-SEM. Herein, the various chitosan-derived porous activated carbon samples are denoted as C-PACt (1:*x*), where *t* is the carbonization temperature and *x* is the activator ratio; the sample prepared without activator is denoted as CS750. Figure 1b shows SEM images of the hierarchical porous carbon samples after carbonization with different activator ratios at 750 °C. In the case of CS750, the as-synthesized cylindrical chitosan flakes show smooth structural features without any porosity (Figure 1b). Interestingly, following introduction of the activator, C-PAC750 (1:0.5) exhibits a hierarchical porous scaffold with a new morphology. Increasing the activator ratio to 1:2 results in a unique hierarchical porous texture, in which the carbon skeleton forms a 3D microporous structure. These structures exhibit randomly opened or broken macropores with sizes varying from a few hundred nanometers to several micrometers that are well interconnected with each other, as shown Figure 1b. However, at a higher activator ratio of 1:3, broken carbon flakes with relatively smooth surfaces and no macro- or mesopores are observed (Figure 1b). The lack of macroscopic porous features on the flake surfaces in C-PAC750 (1:3) can be ascribed to a more violent reaction between carbon and an excess amount of K_2_CO_3_ at the activation temperature. Therefore, via chemical activation, the specific surface areas and hierarchical porous structures of a series of C-PAC750 materials can be adjusted by controlling the chitosan/activator ratio during carbonization. The dependence of the structural features on the chitosan/activator ratio is illustrated in Figure 1a. For comparison, the microstructures of the as-synthesized materials obtained by chitosan carbonization with a constant amount of activator (1:2) at 350, 550, and 750 °C were also observed (Figure S1 in the Supplementary Materials). The morphology of C-PAC350 (1:2) is almost identical to that of chitosan, owing to the low extent of carbonization at an activation temperature of 350 °C. Furthermore, C-PAC550 (1:2) exhibited less porosity than C-PAC750 (1:2). Transmission electron microscopy (TEM) images were recorded to further investigate the microporous structures of the as-prepared C-PAC750 samples, as shown in Figure S2. The TEM images reveal that the series of C-PAC 750 samples have obvious porous structures. All the carbon samples prepared with the activator exhibit large amounts of meso- and micropores.

**Figure 2:**
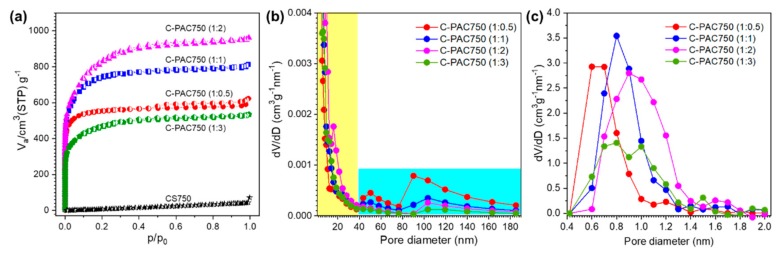
(**a**) N_2_ sorption isotherms of the chitosan-derived carbon samples at 77 K for BET analysis and (**b**) meso- macropore size distribution using the BJH method (**c**) micropore size distribution using MP method of the chitosan-derived carbon samples.

To determine the porosity and surface area of the prepared carbon samples, nitrogen adsorption–desorption experiments were conducted. The nitrogen sorption isotherms of the porous carbons prepared at 750 °C with various chitosan to K_2_CO_3_ ratios are shown in Figure 2a, and the surface areas, total pore volumes, micropore volume and average pore diameters are listed in Table 1. With the exception of the case without the activator (CS750), all the samples exhibit typical Type I isotherms with well-defined plateaus, clearly indicating the microporous character of the porous carbons. Remarkably, C-PAC750 (1:2) showed the highest N_2_ adsorption amount, which can be attributed to the stepwise filling of micro-, meso- and macropores with N_2_ at different relative pressure. Compared with CS750, a K_2_CO_3_/chitosan ratio of 1:2 resulted in a significant increase of the BET(Brunauer-Emmett-Teller) surface area and the total pore volume from 57 to 2850 m^2^·g^−1^ and from 0.09 to 1.48 cm^3^·g^−1^, respectively. However, at higher K_2_CO_3_/chitosan ratios, the surface area decreased. The average pore diameters and BET surface areas of the series of C-PAC750 materials were similar to those reported in the literature [23,24]. These results demonstrate that the introduction of the activator resulted in the generation of macro-, meso-, and micropores in the activated carbons accompanied by significant increases in the BET surface area. The temperature of chitosan carbonization with the activator also strongly affected the features of the produced activated carbons.

**Figure 3:**
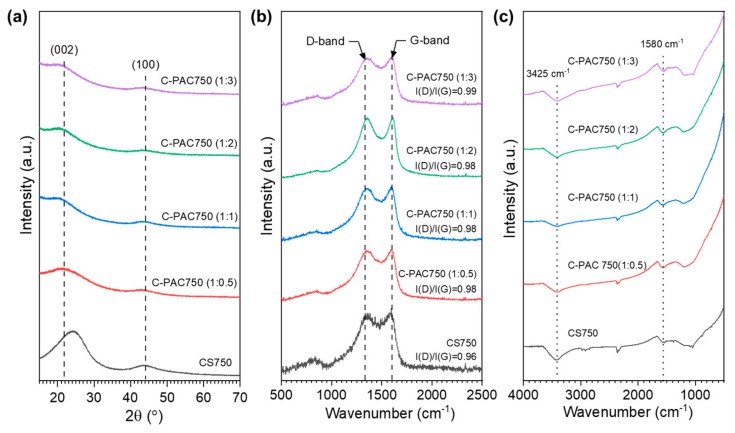
(**a**) XRD pattern (**b**) Raman spectra and (**c**) IR spectra of the chitosan-derived carbon samples.

Figure 2b exhibits the mesopore size distributions of the series of C-PAC750 materials, as calculated by the Barrett-Joyner-Halenda (BJH) method. The highest intensity peak for each sample is observed at a pore diameter of approximately 2.5 nm. Furthermore, C-PAC750 (1:0.5) exhibits more macrospores in the range of 40–190 nm (shaded in blue in Figure 2b) than the other samples, and as the activator ratio increases, the amount of mesopores in this range decreases. In contrast, C-PAC750 (1:2) shows more mesopores in the range below 40 nm (shaded in yellow in Figure 2b) than the other samples. This observation indicates that the activator produces macro- and mesopores and that the distribution of pores is strongly affected by the amount of activator present during chitosan carbonization. Figure 3c exhibits the micropore (MP) plot of series of C-PAC750 materials for the micropore size distributions. As seen, the activator develops micropores in the diameter range from 0.5 to 1.5 nm. In addition, the amount of activator increases, micropores develop and the pore size distribution becomes broader.

The crystalline structures of the carbon samples carbonized with and without the activator were investigated using X-ray powder diffraction (XRD), as shown in Figure 3a. The lack of distinct characteristic peaks in the XRD patterns indicates that all the samples have the typical structure of a disordered carbon material. The two weak broad peaks observed at approximately 23° and 43° correspond to the (002) and (100) reflections of the turbostratic carbon structure, respectively. The broad (100) reflection arises from honeycomb structures formed by sp^2^ hybridized carbons, whereas the broad (002) reflection corresponds to small domains of graphene sheets that exhibit coherent and parallel stacking [25]. Furthermore, the similarity of the XRD patterns of the C-PAC samples suggests that the amount of activator does not affect the structural features. Based on the peak position of the (002) reflection, all the C-PAC samples prepared with the activator have the same d-spacing value of 0.386 nm.

To investigate the surface crystal structures in the series of C-PAC750 materials, a Raman analysis was performed. As shown Figure 3b, all the samples exhibit two distinct bands, namely, the D-band at approximately 1350 cm^−1^ and the G-band at approximately 1580 cm^−1^. Generally, intensities of the D-band and G-band indicate the concentration of disordered carbon and the concentration of graphitized carbon, respectively [26]. For all the samples, the intensity of the G band is slightly higher or similar to that of the D band, indicating a high extent of graphitization. The intensity ratio of these bands, I(D)/I(G), can be used to estimate the disordered carbon content in carbon specimens. As seen in Figure 3b, changing the activator/chitosan ratio had little effect on the I(D)/I(G) ratio because the amount of activator affects the appearance of the sample but does not affect the degree of carbonization.

To identify the main surface groups, FT-IR spectra of the series of C-PAC750 materials were recorded, as shown in Figure 3c. A broad absorption band at approximately 3425 cm^−1^ corresponding to N–H or O–H stretching vibrations was observed for all the C-PAC750 samples. The peak at approximately 1580 cm^−1^ corresponds to the N–H in-plane deformation vibrations or C=C stretching vibrations of aromatic rings. The broad peak at 1200–1000 cm^−1^ is attributed to C–N or C–O stretching vibrations. Therefore, the FT-IR spectra are consistent with the presence of N–H and C–N species in all the C-PAC750 materials.

**Figure 4:**
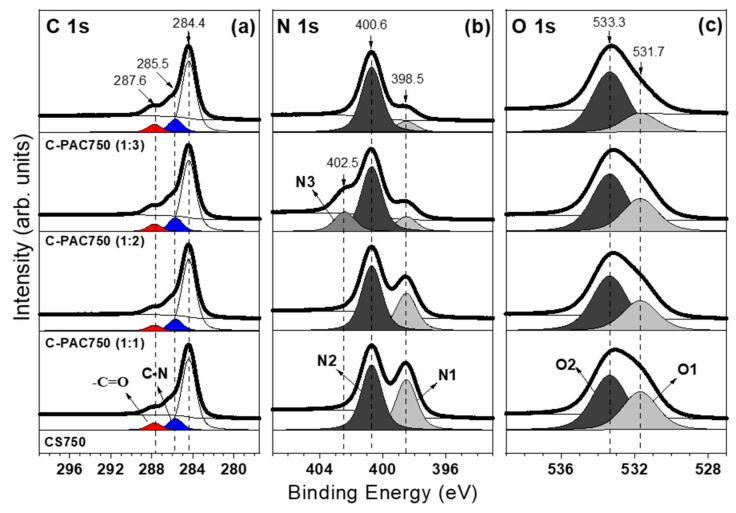
(**a**) C 1s, (**b**) N 1s, and (**c**) O 1s core-level spectra for the chitosan-derived carbon samples.

The chemical states of C, O, and N species in the series of C-PAC750 samples were systemically examined using X-ray photoelectron spectroscopy (XPS). Deconvolution of the high-resolution C 1s core-level spectra (Figure 4a) gave three peaks with binding energies of approximately 284.4, 285.5, and 287.6 eV corresponding to sp^2^ C–H, C–N, and –C=O species, respectively, where –C=O refers to various oxygen-containing functional groups, including –C=O, and –COOH [27]. The spectra changed slightly depending on the amount of activator and in the case of C-PAC750 (1:3), only the content of C–N groups increased slightly. These observations indicate that the degree of chitosan carbonization was similar regardless of the amount of activator at 750 °C, but changes occurred related to the nitrogen- and oxygen-containing functional groups in the carbon framework.

To clarify how the chemical states of nitrogen and oxygen changed with the amount of activator, the N 1s (Figure 4b) and O 1s (Figure 4c) core-level spectra were analyzed [28,29]. Deconvolution of the high-resolution N 1s spectra (Figure 4b) gave two individual peaks at binding energies of 398.5 and 400.6 eV, assigned to NH_2_ (amine; N1) and NH_3_^+^ (dative amine; N2), respectively, which were produced by two nitrogen-containing functional groups during the carbonization process. Clearly, as the amount of activator increases, the N2 peak tends to increase relatively to N1 and the amount of nitrogen in the carbon framework decreases. For example, the amount of nitrogen in C-PAC750 (1:3) is decreased by 20% compared with that in C-PAC750 (1:1). For C-PAC750 (1:2), a new peak appears, which is assigned to NO_2_ (labeled as N3).

The high-resolution O 1s spectra (Figure 4c) show two peaks at 531.7 and 533.3 eV, assigned to OH (labeled O1) and C–O–C (labeled O2), respectively [29,30]. The spectra do not change significantly from CS 750 to C-PAC750 (1:2), but a significant reduction in the intensity of the two peaks is observed in the spectrum of C-PAC750 (1:3). Thus, the XPS analysis confirms that that excess activator effectively removes reactive groups, which will affect the adsorption and desaturation properties.

### 3.2. Evalulation of DMMP Adsorption Properties of C-PAC Samples

**Figure 5:**
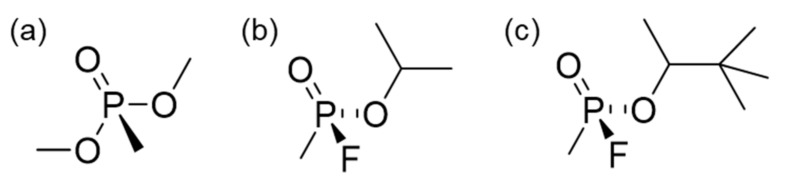
(**a**) Dimethyl methylphosphonate (DMMP), (**b**) Sarin (GB), and (**c**) Soman (GD).

Owing to safety reasons, the CWA simulant, DMMP, is widely used to investigate the sorption properties of G-series nerve agents, such as sarin (GB) and soman (GD), which possess similar functional groups (Figure 5). In particular, DMMP is generally employed as an adsorption simulant because it does not have the highly reactive P–F group common to all G-agents. Many theoretical and experimental studies on the adsorption of G-series nerve agents show that DMMP adsorption onto different types of adsorbent, such as carbon materials [31], metal oxides [32], and zeolites [33], occurs through the phosphoryl group. XPS spectra of the C-PAC750 materials before and after DMMP adsorption were almost identical (Figure S3 in the Supplementary Materials). This observation indicates that DMMP does not chemisorb into these materials but rather is physiosorbed on the surface.

We evaluated the ability of the C-PAC750 materials prepared with different activators to chitosan ratios under ambient conditions. Typically, a certain amount of carbon sample was kept in a desiccator with a certain amount of DMMP (0.5 g·L^−1^) for a specific period of time. Subsequently, to determine the extent of adsorption, each sample was subjected to a desorption process and desorbed DMMP was analyzed quantitatively by GC. Simultaneously, the amount of DMMP adsorbed onto the carbon samples was determined by TGA.

**Figure 6:**
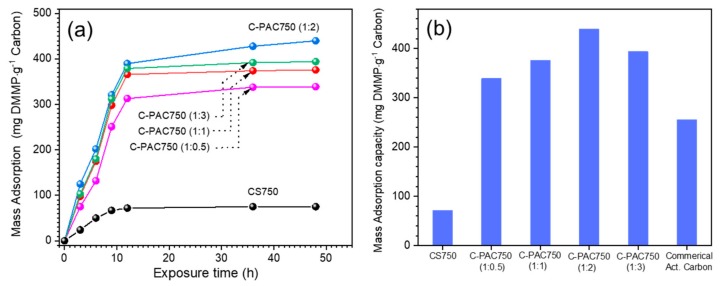
(**a**) DMMP uptake as a function of time and (**b**) mass adsorption capacity after 48 h for the chitosan-derived carbon samples.

The amounts of DMMP adsorbed on the C-PAC750 materials over time are plotted in Figure 6a. The adsorption curves of all the samples exhibit a similar trend with an initial rapid increase over the first 36 h, followed by saturation, which is consistent with a typical adsorption process. After 48 h, C-PAC750 (1:2) had the highest DMMP uptake, followed by C-PAC750 (1:3), C-PAC750 (1:1), and C-PAC750 (1:0.5). Furthermore, nonporous CS 750, which was carbonized without any activator, exhibited a very low adsorption capacity for DMMP. To probe the adsorption regime between full surface coverage and liquid saturation, we evaluated the samples after 48 h of DMMP exposure (Figure 6b). In particular, the DMMP adsorption capacity of C-PAC750 (1:2) was saturated at approximately 412 mg·g^−1^ carbon after 48 h. This relationship between DMMP uptake and total surface area is similar to that observed from other reports.

To demonstrate the advantages of C-PAC750 (1:2) for adsorbing DMMP, the DMMP adsorption capacity of a representative commercial activated carbon is also displayed in Figure 6b. Activated carbon is considered to have excellent adsorption properties for the removal of real agents, such as sarin and soman, in the field. However, the DMMP adsorption capacity of activated carbon is only 212 mg·g^−1^ carbon, which is approximately 50% of that of C-PAC750 (1:2) under our conditions.

## 4. Conclusions

In this study, porous carbon materials with various porosities and surface areas were synthesized by controlling the activator to chitosan ratio. Furthermore, the characteristics of the C-PAC materials as adsorbents for DMMP removal were examined. The C-PAC materials showed high adsorption capacities owing to the affinity between the carbon surface and DMMP molecules and the adsorption capacities improved as the surface area increased. The best adsorption characteristics were observed when the activator/chitosan ratio was 1:2, with a DMMP adsorption capacity that was two times higher than that of commercial activated carbon. This study revealed that chitosan, which is a natural waste biopolymer, is a good precursor for porous activated carbons. Furthermore, the results suggest that C-PAC materials are feasible adsorbents for DMMP. Thus, C-PAC750 (1:2) is advantageous for use in protective gear, such as masks, suits, gloves, and cleaning mats, for a variety of protective or remedial applications.

## Figures and Tables

**Table 1:** Porous structural parameters of chitosan-derived carbon samples.

Samples	S_BET_^a^ (m^2^·g^−1^)	V_tot_^b^ (cm^3^·g^−1^)	V_micro_^c^ (cm^3^·g^1^)	d ^d^ (nm)
CS750	57	0.09	0.03	0.64
C-PAC750 (1:0.5)	2084	0.96	0.88	1.84
C-PAC750 (1:1)	2643	1.26	1.20	1.90
C-PAC750 (1:2)	2850	1.48	1.41	2.08
C-PAC750 (1:3)	1639	0.82	0.79	2.01

^a^ BET surface area, ^b^ total pore volume at *p*/*p*_0_ = 0.99, ^c^ micropore volume by the t-plot method and ^d^ average pore diameter.

## Supplementary Materials

The following are available online at https://www.mdpi.com/2079-4991/9/12/1703/s1, Figure S1: SEM images, Figure S2: TEM, Figure S3: XPS spectra.
