# Supplementary material for: Chitosan-Derived Porous Activated Carbon for the Removal of the Chemical Warfare Agent Simulant Dimethyl Methylphosphonate
Source: Nanomaterials (Basel). 2019 Nov 28;9(12):1703. doi: 10.3390/nano9121703 (PMC6956094; doi:10.3390/nano9121703)
Supplement: Supplementary file 1 [file nanomaterials-09-01703-s001.pdf]

Supplementary Materials

Chitosan-Derived Porous Activated Carbon for the Removal of the Chemical Warfare Agent Simulant Dimethyl Methylphosphonate

Hyejin Yu [1,2], Ye Rim Son [1], Hyeonji Yoo [1], Hyun Gil Cha [2,*], Hangil Lee [3,*] and Hyun Sung Kim [1,*]

[1] Department of Chemistry, Pukyong National University, Busan 48513, Korea; yuhj93@krict.re.kr (Hyejin Yu); syl75218@daum.net (Y.R.S.); hjyoo1996@gmail.com (Hyeonji Yoo)

[2] Center for Bio-based Chemistry, Korea Research Institute of Chemical Technology (KRICT), Ulsan 44429, Korea

[3] Department of Chemistry, Sookmyung Women's University, Seoul 04310, Korea

* Correspondence: hgcha@krict.re.kr (H.G.C.); easyscan@sookmyung.ac.kr (H.L.); kimhs75@pknu.ac.kr (H.S.K.); Tel.: +82-51-629-5596 (H.S.K.)

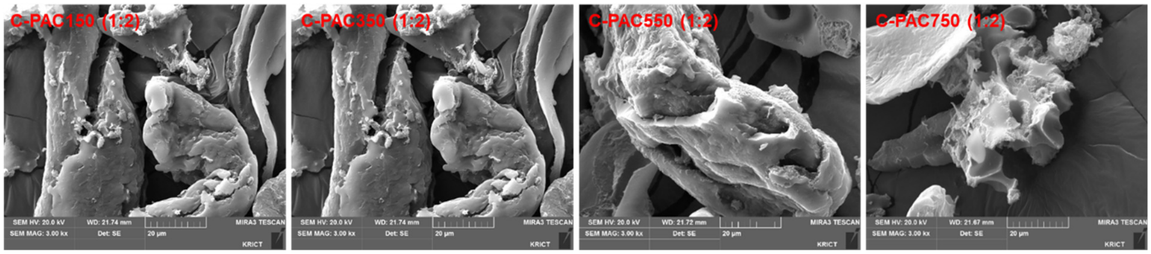

Figure S1. SEM images of activated carbons prepared by carbonization of chitosan with chitosan and activator (1:2) ratio at various temperature as indicated.

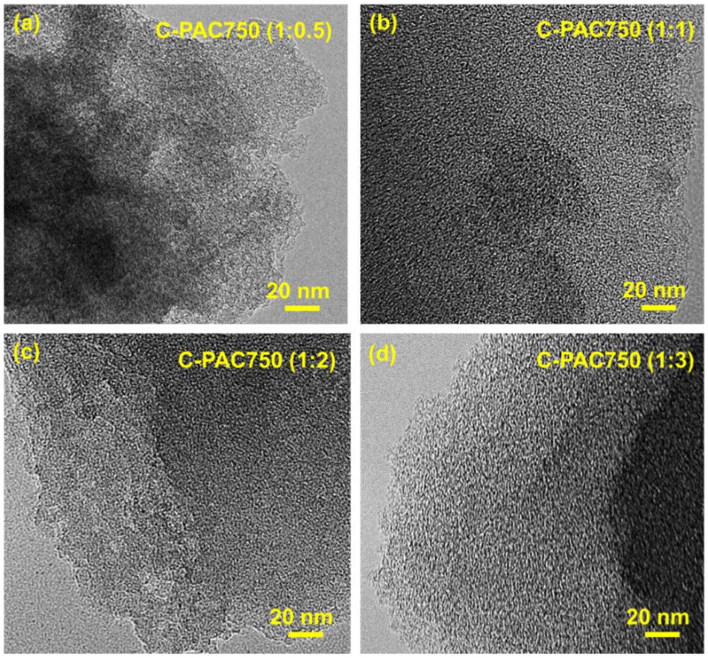

Figure S2. TEM images of the series of C-PAC750 materials as indicated.

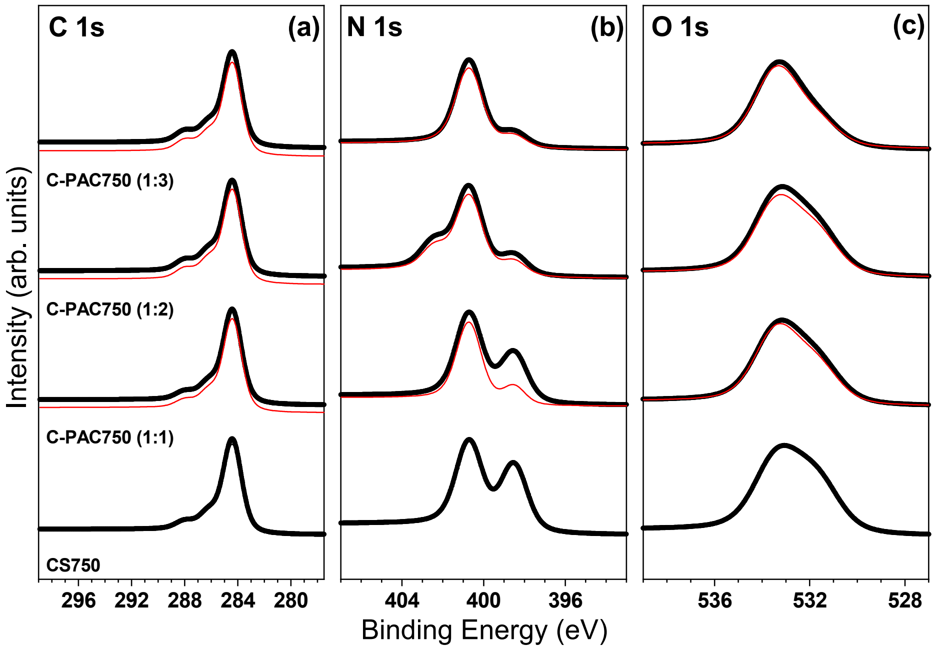

Figure S3. (**a**) C 1s, (**b**) N 1s, and (**c**) O 1s XPS spectra for the chitosan-derived carbon samples before and after adsorption of DMMP.
